# Identification and Expression Profile of Chemosensory Genes in the Small Hive Beetle *Aethina tumida*

**DOI:** 10.3390/insects12080661

**Published:** 2021-07-21

**Authors:** Lixian Wu, Xin Zhai, Liangbin Li, Qiang Li, Fang Liu, Hongxia Zhao

**Affiliations:** 1 Guangdong Key Laboratory of Animal Conservation and Resource Utilization, Guangdong Public Laboratory of Wild Animal Conservation and Utilization, Institute of Zoology, Guangdong Academy of Sciences, Guangzhou 510260, China; lixianwu@giz.gd.cn (L.W.); xinleioli@163.com (X.Z.); 19991111@stu.scau.edu.cn (L.L.); 18434763376@163.com (Q.L.); 2 College of Plant Protection, South China Agricultural University, Guangzhou 510642, China; 3 College of Animal Science, Shanxi Agricultural University, Taigu 030801, China

**Keywords:** *Aethina tumida*, transcriptome, chemosensory genes, identification, expression analysis

## Abstract

**Simple Summary:**

The small hive beetle is a destructive pest of honeybees, causing severe economic damage to the apiculture industry. Chemosensory genes play key roles in insect behavior, such as foraging and mating partners. However, the chemosensory genes are lacking in the small hive beetle. In order to better understand its chemosensory process at the molecular level, a total of 130 chemosensory genes, including 38 odorant receptors, 24 ionotropic receptors, 14 gustatory receptors, 3 sensory neuron membrane proteins, 29 odorant binding proteins, and 22 chemosensory proteins were identified from the transcriptomic data of antennae and forelegs. Reverse-transcription PCR showed that 3 OBPs (*AtumOBP3*, 26 and 28) and 3 CSPs (*AtumCSP7*, 8 and 21) were highly expressed in antennae. Overall, this study could provide a basis for elucidating functions of these chemosensory genes at the molecular level.

**Abstract:**

*Aethina tumida* is a parasite and predator of honeybee causing severe loss to the bee industry. No effective and environmentally friendly methods are available to control this pest at present. Chemosensory genes play key roles in insect behavior which can potentially be used as targets for developing environmentally friendly pest control agents. In this study, the putative chemosensory genes in antennae and forelegs of *A. tumida* involved in olfaction or contact chemical communication of adults were investigated using RNA transcriptome sequencing and PCR methods. Based on transcriptomic data, unigenes encoding 38 odorant receptors (ORs), 24 ionotropic receptors (IRs), 14 gustatory receptors (GRs), 3 sensory neuron membrane proteins (SNMPs), 29 odorant binding proteins (OBPs), and 22 chemosensory proteins (CSPs) were identified. The analyses of tissue expression profiles revealed that genes encoding 38 ORs, 13 antennal IRs, 11 GRs, 1 SNMP, 24 OBPs, and 12 CSPs were predominately expressed in antennae. No significant differences in expression levels of these genes were found between males and females. Genes encoding 5 non-NMDA iGluRs, 3 GRs, 2 SNMPs, 5 OBPs, and 12 CSPs were predominately expressed in forelegs. RT-PCR assays for SNMPs, OBPs and CSPs further revealed that 3 OBPs (*AtumOBP3*, 26 and 28) and 3 CSPs (*AtumCSP7*, 8 and 21) were highly expressed in antennae. Our results enrich the gene inventory of *A. tumida* and facilitate the discovery of potential novel targets for developing new pest control measures.

## 1. Introduction

Insects rely on olfaction to recognize and discriminate chemical cues during foraging, mating, and oviposition [1,2]. The perception of chemical cues (general odors and pheromones) starts with the detection of volatile molecules at insect antennae, which served as an important periphery olfactory system [3], a critical signaling process that involves multiple proteins, including odorant receptors (ORs), ionotropic receptors (IRs), gustatory receptors (GRs), sensory neuron membrane proteins (SNMPs), odorant binding proteins (OBPs), and chemosensory proteins (CSPs) [2,4,5,6].

Insect chemoreceptors consist of ORs, IRs and GRs are located in the dendritic membrane of olfactory receptor neurons (ORNs). ORs are receptors with two heteromeric subunits and each subunit with seven-transmembrane domains. ORs are composed of a highly conserved, universal co-receptor (Orco; formerly called OR83b) and a variable partner (named ORX) that interacts with specific ligands [7]. IRs are a subfamily of ancient and highly conserved ionotropic glutamate receptors (iGluRs) with atypical binding domains, which form ligand-gated cation channels [4]. Insect IRs are generally divided into two subgroups: one is “antennal IRs”, expressed in insect antennal ORNs and involved in olfaction, gustation, thermosensation and hygrosensation; the other is species-specific “divergent IRs,” mainly expressed in the gustatory organs and appear to be absent from antennae [8]. GRs are mainly expressed in gustatory receptor neurons in taste organs and are associated with contact chemoreception. However, some GRs such as carbon dioxide receptors and sugar receptors, are also expressed in antennal dendrites among various insects [9,10]. SNMPs, which belong to the CD36 protein family, are located in the dendritic membranes of pheromone sensitive neurons and have roles in pheromone recognition [11,12,13,14]. OBPs are soluble proteins that solubilize and bind hydrophobic odorant molecules from the external environment and transfer them to chemosensory receptors embedded in ORNs [2,15]. CSPs are a family of small soluble proteins that are abundant in the sensillar lymph [16]. The exact roles of CSPs in olfactory transduction remain largely unknown. In some insect species, antenna-predominant CSPs exhibit binding activity with plant volatiles and pheromones with similar functions to OBPs [17,18,19,20].

The small hive beetle *Aethina tumida* Murray (Coleoptera: Nitidulidae) is a destructive pest of honeybees. The beetle feeds on bee nest products and has serious negative impact on honeybees and other pollinators. The small hive beetle has spread to Sub-Saharan Africa and is now found in all continents except Antarctica [21,22,23]. In addition to honeybees, the small hive beetle is also a pest of bumblebees and stingless bees. This beetle pest poses a serious threat to the whole global honeybee industry. To control this emerging pest, Neumann and Ellis have proposed to develop trapping systems using an *A. tumida* pheromone [24]. Recently, an aggregation pheromone comprised of 6-methyl-5-hepten-2-one, nonanal and decanal has been identified with potential for controlling *A. tumida*. Also, *A. tumida* is highly attracted to volatiles emitted by adult honeybees (*Apis mellifera*), bumble bees (*Bombus impatiens*), stored pollen, wax, brood, and honey [25,26,27]. Therefore, olfaction-based approaches using aggregation pheromones or host attractants, would be developed as an environmentally friendly strategy against this destructive pest. Additionally, the peripheral olfactory proteins, including ORs and OBPs, are potential targets for designing super-ligands and screening semichemicals for pest management [28,29,30]. Towards this direction, the molecular mechanism for *A. tumida* to perceive these volatiles needs to be revealed.

The objective of this study was to identify the repertoire of chemosensory genes (ORs, IRs, GRs, SNMPs, OBPs, and CSPs) of the small hive beetle via a transcriptome analysis. We performed transcriptomes from dissected antennae and forelegs from both male and female adults. Insect forelegs are involved in sensing non-volatile chemicals after insects landing on the host [31,32]. Based on the differences in expression levels of chemosensory genes between antennae and forelegs of both males and females, candidate chemosensory genes that might be involved in olfaction and gustation were identified. Phylogenetic analyses were also conducted with identified proteins in conjunction with homologues from other Coleopterans. This work provides valuable data for further functional studies of these chemosensory genes in the small hive beetle at the molecular level.

## 2. Materials and Methods

### 2.1. Insects and Tissue Collections

A small hive beetle colony was maintained in Institute of Zoology, Guangdong Academy of Sciences in Guangzhou city and was originally established from a sample collected from naturally infested colonies of *Apis cerana cerana* in the Shanwei city, Guangdong province, China (115°33′ E, 23°11′ N). The colony was maintained at 20 °C, 65% RH, under darkness.

For transcriptome sequencing, 300 pairs of antennae and forelegs were dissected separately from females and males of the small hive beetle. Three replicates were included for each transcriptomic analysis. For RT-PCR analyses, we dissected the different tissues including the antennae, forelegs, wings, and genitals from adults (male: female = 1:1, *n* = 80 each). Tissue samples were homogenized to powder immediately in liquid nitrogen and stored at −80 °C until RNA extraction.

### 2.2. RNA Extraction, cDNA Library Construction, Illumina Sequencing and De Novo Assembly

Total RNA was extracted using Trizol reagent (Takara, Kyoto, Japan) and potential genomic DNA contamination was removed by RNase-free DNaseI (Invitrogen, Waltham, MA, USA). RNA integrity was examined via agarose gel electrophoresis, and RNA purity and concentration were assessed using a NanoDrop spectrophotometer (Wilmington, NC, USA).

One microgram purified RNA per sample was used as input material for library construction. Libraries were constructed using an Illumina’s TruSeq RNAseq Sample Prep kit (Illumina, CA, USA) following the manufacturer’s instruction. cDNA libraries were evaluated using an Agilent 2100 Bioanalyzer, and sequenced on the Illumina HiSeq 4000 platform (Illumina, CA, USA) with 150 bp paired-end module (Novogene, Beijing, China). Raw reads were firstly processed by removing adaptor sequences, unknown (poly-N) and low-quality reads and subsequently assembled into unigenes using Trinity (version: 2.0.6) Software (Broad Institute, MA, USA) with default parameters.

### 2.3. Functional Annotation and Chemosensory Gene Identification

Gene annotation was achieved by searching these unigenes against the NCBI non-redundant protein database (NR), Swiss-Prot (http://www.ebi.ac.uk/uniprot, accessed on 10 December 2019), Cluster of Orthologous Groups of proteins (COG) and Gene Ontology (GO) databases using BlAST program (E-value < 1 × 10^−5^). To identify unigenes coding for ORs, IRs, GRs, SNMPs, OBPs, and CSPs, known protein sequences from other Coleopteran species (Supplementary Table S1) were selected as queries to search the antennae and forelegs transcriptomes of *A. tumida*. tBLASTn was also used to search and identify candidate gene, with an E-value cut of 10^−5^. Candidate genes were rechecked using BLASTx against protein databases at NCBI (http://www.ncbi.nlm.nih.gov/, accessed on 14 December 2019). Open reading frame (ORF) of candidate chemosensory genes were predicted using the ORF Finder in NCBI (https://www.ncbi.nlm.nih.gov/orffinder/, accessed on 14 December 2019). The conserved domain, signal peptide and cysteine location in candidate genes were analyzed by using the InterProScan tool plug-in in Geneious (Reachsoft, Beijing, China) [33]. Candidate unigenes coding for ORs, IRs, GRs, SNMPs, OBPs, CSPs, and reference genes were listed in Supplementary Table S2.

### 2.4. Sequence and Phylogenetic Analyses

The amino acid sequences of candidate chemosensory genes were aligned using Clustal Omega [34]. Phylogenetic trees were generated using FastTree2 with the Maximum-likelihood methodJones-Taylor-Thornton amino acid substitution model [35]. Node support was assessed by bootstrap analyses of 1000 replicates. Phylogenetic trees were visualized using FigTree (http://tree.bio.ed.ac.uk/software/figtree, accessed on 11 December 2019). The data set contained genes identified in other Coleopterans as follows: 257 OR sequences from the OR data set (31 from *Colaphellus bowringi*, 54 from *Cylas formicarius*, 32 from *Dendroctonus ponderosae*, 48 from *Megacyllene caryae* and 92 from *Tribolium castaneum*); 87 IR sequences from the IR data set (18 from *Basilepta melanopus*, 19 from *Brontispa longissima*, 15 from *C. formicarius*, and 35 from *T. castaneum*); 278 GR sequences from the GR data set (16 from *B. melanopus*, 11 from *C. formicarius*, 68 from *Drosophila melanogaster*, 2 from *D. ponderosae* and 181 from *T. castaneum*; 16 SNMP sequences from the SNMP data set (4 from *B. melanopus*, 3 from *C. formicarius*, 2 from *D. melanogaster*, 3 from *D. ponderosae*, 2 from *Phyllotreta striolata* and 2 from *T. castaneum*; 87 OBP sequences from the IR data set (26 from *C. bowringi*, 50 from *T. castaneum* and 11 from other Coleopterans); 74 CSP sequences from the CSP data set (19 from *B. melanopus*, 12 from *C. bowringi*, and 12 from *C. formicarius*, 11 from *D. ponderosae*, and 20 from *T. castaneum*). The data sets of chemosensory genes chosen from other insect species are listed in Supplementary Table S3.

### 2.5. Transcript Abundance of Chemosensory Genes

To estimate the expression levels of the candidate chemosensory genes in female antennae (FA), male antennae (MA), female forelegs (FL), and male forelegs (ML), the average FPKM (fragments per kilobase of exon per million fragments mapped) values were used [36]. The estimated expression levels of chemosensory genes are listed in Supplementary Table S4. Heatmaps of gene expression for different chemosensory genes among the antennae and forelegs of female and male were generated by R (version: 3.4.1) (Bioconductor, MA, USA). Differentially expressed genes were identified between FA and MA using DESeq2 (version: 1.6.3) (Bioconductor, MA, USA) [37]. 

### 2.6. RT-PCR

Reverse-transcription PCR (RT-PCR) was employed to examine the expression pattern of 29 OBPs, 22 CSPs, and 3 SNMPs in different tissues including antennae, foreleg, wings, and genitals. Total RNA was extracted from these tissues, and the first-strand cDNA was synthesized by using a HiScrip III RT SuperMix Kit (Vazyme, Nanjing, China). PCR was performed under the following conditions: 95 °C for 2 min, followed by 30 cycles of 95 °C for 30 s, 56 °C for 30 s, 72 °C for 1 min, and a final extension for 10 min at 72 °C. *GAPDH* and *E-cadherin* were used as internal reference genes. The PCR products were subjected to electrophoresis and the results were analyzed by gel imaging (Tanon, Shanghai, China). At least three independent biological replicates were performed in this analysis. The gene-specific primers were designed using Primer5 software [38], and they were listed in Supplementary Table S5.

## 3. Results

### 3.1. Overview of A. tumida Transcriptomes

We sequenced the transcriptomes of female antennae (FA), male antennae (MA), female forelegs (FL), and male forelegs (ML) of A. tumida with three independent biological replicates. We obtained approximately 50.06 (FA1), 56.84 (FA2), 63.25 (FA3), 57.71 (MA1), 56.14 (MA2), 52.61 (MA3), 54.15 (FL1), 48.96 (FL2), 47.51 (FL3), 53.76 (ML1), 44.18 (ML2), and 48.87 (ML3) million clean reads from these samples. Clean reads were assembled into 34,531 unigenes with an average length of 1430 bp, and an N50 of 2403 bp (Supplementary Table S6). The datasets of transcriptomes during the current study have been submitted to the NCBI’s Sequence Read Archive database (BioProject Accession Number: PRJNA596813).

After annotation, there were 21,775 (63.05%), 15,912 (46.08%), 19,365 (56.08%), 7705 (22.31%), 19,561 (56.64%), 19,561 (56.64%), and 10,247 (29.67%) unigenes that homologous sequences were found in NCBI-nr, NCBI-nt, Swiss-Prot, KO, PFAM, GO and KOG databases, respectively. The overall 26,877 (77.83%) unigenes were annotated according to the homologous sequences (Supplementary Table S6).

### 3.2. Candidate Genes Coding for ORs

**Figure 1:**
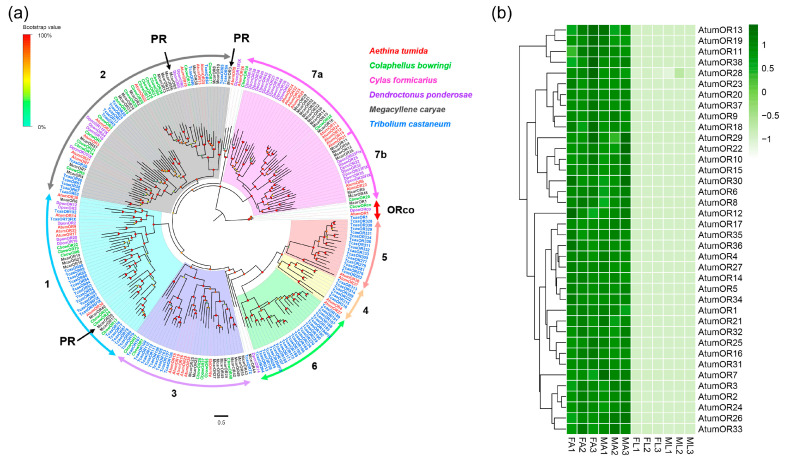
Analyses of putative odorant receptors (ORs) in *A. tumida*. (**a**) Maximum-likelihood tree of putative ORs. The tree was rooted with the conserved Orco orthologues. Circles at the branch nodes represent bootstrap values based on 1000 replicates. Scale bar represents the 0.5 amino acid substitutions per site. The different subfamilies are highlighted as blue, gray, purple, yellow, coral, green and pink, respectively. Orco is highlighted as red. (**b**) Heatmap of ORs based on antennae and forelegs transcriptome data in *A. tumida*. FA, female antennae; MA, male antennae; ML, male forelegs; FL, female forelegs. The transcript abundance was measured by FPKM-values. All 38 ORs were mainly expressed in the FA and MA. Three independent biological replicates were conducted for each sample (such as FA1, FA2, and FA3).

In total, 38 putative OR-encoding unigenes (*AtumOR1-38*) were identified based on the combined transcriptome data from *A. tumida* (Supplementary Table S2: Sheet 1). Of these, 19 unigenes encode the full-length proteins of 300–400 amino acids with 2–8 transmembrane domains (TMDs). Meanwhile, five AtumORs (AtumOR1, 2, 7, 35 and 36) encoded seven-TMDs (see Supplementary Table S2: Sheet 1). Furthermore, we identified an OR gene (*AtumOR1*) had a high sequence homology with the conserved insect *Orco* gene family and named it *AtumOrco*. In previous studies, phylogenetic analysis has separated ORs in Coleopteran species apart from the *Orco* gene subgroup (which includes *AtumOrco*, *CbowOrco*, *DponOrco* and *TcasOrco*), into multiple subgroups numbered 1–7 [39,40] (Figure 1a). Following the maximum-likelihood phylogenetic analysis, except two AtumORs (*AtumOR8* and 26), 36 ORs were divided into six subgroups (group 1–5, and 7), with six ORs assigned to group 1, eight ORs assigned to group 2, six ORs assigned to group 3, two ORs assigned to group 4, two ORs assigned to group 5, and 11 sequences assigned to group 7, respectively. Group 7 was further assorted into two subsets: group 7a and group 7b. The rest one subgroup 6 contained only *T. castaneum* ORs. No AtumORs were clustered with high homology to known functional ORs, such as pheromone receptors from *M. caryae* [40]. 

The expression levels of genes encoding all 38 ORs were assessed using FPKM-values (Figure 1b). Our transcriptome analysis showed that all 38 ORs were expressed in antennae (ranged from 0.8 to 41.4, mean FPKM). No gene was expressed in forelegs (Supplementary Table S4). The *AtumOR1* (*Orco*) had the highest level of expression in antennae from both males and females (FA: 41.4, MA: 38.6, mean FPKM), followed by *AtumOR24* (FA: 11.7, MA: 11.6, mean FPKM). Genes encoding all 38 ORs exhibited similar expression patterns in the FA and MA.

### 3.3. Candidate Coding Genes for iGluRs/IRs

**Figure 2:**
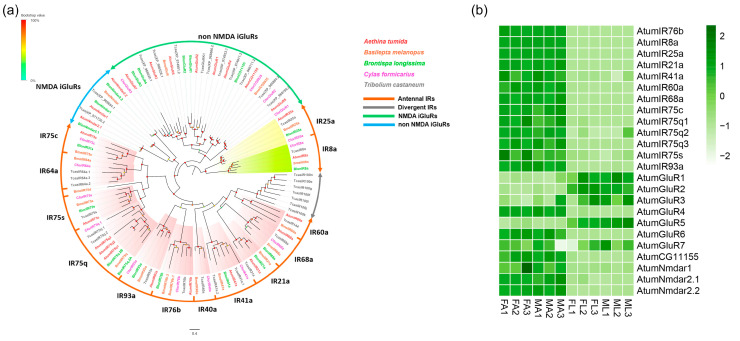
Analyses of putative ionotropic recptors (IRs) in *A. tumida*. (**a**) Maximum-likelihood tree of putative IRs. The tree was rooted with the conservative IR8a/IR25a orthologues. Circles at the branch nodes represent bootstrap values based on 1000 replicates. Scale bar represents the 0.4 amino acid substitutions per site. Antennal IRs are marked with orange; Divergent IRs are marked with gray; NMDA iGluRs are marked with green; non-NMDA iGluRs are marked with blue. (**b**) Heatmap of IRs based on antennae and forelegs transcriptome data in *A. tumida*. FA, female antennae; MA, male antennae; ML, male forelegs; FL, female forelegs. The transcript abundance was measured by FPKM-values.

Twenty-four putative iGluR/IR-encoding genes were identified (Supplementary Table S2: Sheet 2). Among them, 23 iGluRs/IRs had full-length ORFs, with at least 369 amino acid residues. All putative proteins contained at least a ligand binding domain (LBD) or a Lig_Chan domain, which are characteristics of most insect IRs. According to the phylogenetic analysis of iGluRs/IRs from five Coleopteran species, all the identified iGluRs/IRs can be classified into different subgroups, including (N-Methyl-D-aspartic acid) NMDA iGluRs, non-NMDA iGluRs, antennal IRs, and divergent IRs (Figure 2a). A group of “antennal IR” conserved among Coleopterans was also observed. Thirteen AtumIRs, including *AtumIR8a*, *21a*, *25a*, *41a*, *60a*, *68a*, *75c*, *75q1*, *75q2*, *75q3*, *75s*, *76b*, and *93a*, were clustered with their orthologs. Genes encoding all antennal IRs from *A. tumida* were expressed at relatively high levels in both FA and MA. In comparison, genes encoding a number of non-NMDA iGluRs (*AtumGluR1*, *2*, *3*, *5,* and *7*) were expressed at higher levels in FL and ML (Figure 2b). The gene encoding the co-receptor *AtumIR76b* had the highest expression (FA: 114.3, MA: 108.1, mean FPKM) among all antennal IRs of the small hive beetle, followed by *AtumIR8a* (FA: 42.5, MA: 41.9, mean FPKM) and *AtumIR25a* (FA: 23.2, MA: 21.8, mean FPKM) (Supplementary Table S4).

### 3.4. Candidate Genes Coding for GRs

**Figure 3:**
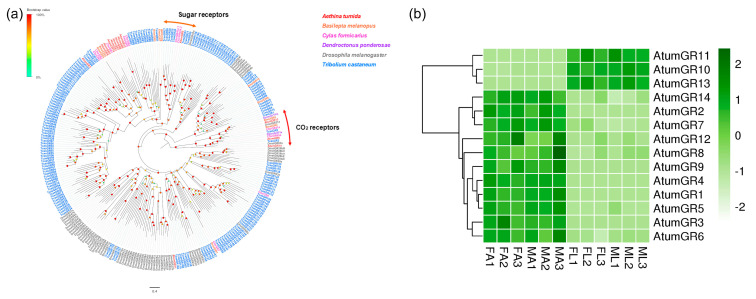
Analyses of putative gustatory receptors (GRs) in *A. tumida*. (**a**) Maximum-likelihood tree of putative GRs. The distance tree was rooted by the conservative GR21a/GR63a orthologues. Circles at the branch nodes represent bootstrap values based on 1000 replicates. Scale bar represents the 0.4 amino acid substitutions per site. (**b**) Heatmap of GRs based on antennae and forelegs transcriptome data in *A. tumida*. FA, female antennae; MA, male antennae; ML, male forelegs; FL, female forelegs. The transcript abundance was measured by FPKM-values.

Fourteen putative GR-encoding genes (*AtumGR1-14*) were identified (Supplementary Table S2: Sheet 3). Among these GR genes, *AtumGR5*, *6*, *7*, *10*, *12*, and *13* contained full length open reading frames, which encode putative proteins with 279 to 461 amino acids. A phylogenetic tree was built with GRs from *A. tumida* and other Coleopterans as well as *D. melanogaster* (Figure 3a). Proteins encoded by *AtumGR6*, *13*, and *14* were grouped with *Drosophila* carbon dioxide receptors *DmelGR21a* and *DmelGR63a* [8], indicating that these genes were responsible for carbon dioxide sensing. *AtumGR5* clustered within *DmelGR43a* [41], which has been shown to detect sugars in *D. melanogaster*. In addition, most of the remaining AtumGRs were assigned to two phylogenetic group with GRs of *B. melanopus* and *C. formicarius*. In addition, 11 AtumGRs were relatively high in the FA and MA, while two AtumGRs (*AtumGR10* and *13*) were more highly expressed in the FL and ML (Figure 3b). The expression of the genes encoding putative carbon dioxide receptor (*AtumGR14*) was the highest in antennae. The expression of a gene encoding an unknown GR (*AtumGR10*) was the highest in forelegs (Supplementary Table S4).

### 3.5. Candidate Genes Coding for SNMPs

**Figure 4:**
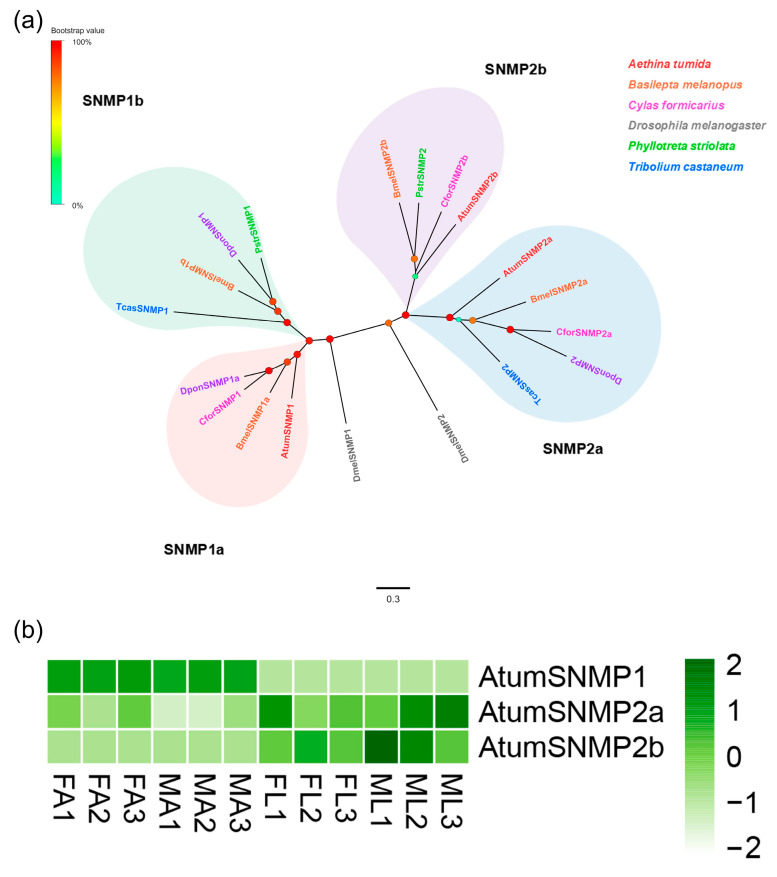
Analyses of putative sensory neuron membrane proteins (SNMPs) in *A. tumida*. (**a**) Maximum-likelihood tree of putative SNMPs. Circles at the branch nodes represent bootstrap values based on 1000 replicates. Scale bar represents the 0.3 amino acid substitutions per site. The different clades are highlighted as pink, blue, green and purple, respectively. (**b**) Heatmap of SNMPs based on antennae and forelegs transcriptome data in *A. tumida*. FA, female antennae; MA, male antennae; ML, male forelegs; FL, female forelegs. The transcript abundance was measured by FPKM-values.

Three putative SNMP-encoding genes were identified, which contained full-length ORFs and two TMDs (Supplementary Table S2: Sheet 4). Four distinct groups, namely *SNMP1a*, *SNMP1b*, *SNMP2a,* and *SNMP2b*, were observed in a phylogenetic tree generated with our identified sequences and paralogs from other Coleopterans and *D. melanogaster* (Figure 4a). AtumSNMP1, 2a and 2b were classified into *SNMP1a*, *SNMP2a,* and *SNMP2b* groups, respectively. In terms of expression, *AtumSNMP1* was expressed at relatively high levels in FA and MA, while *AtumSNMP2a* and *2b* expressed highly in the FL and ML (Figure 4b).

### 3.6. Candidate Genes Coding for OBPs

**Figure 5:**
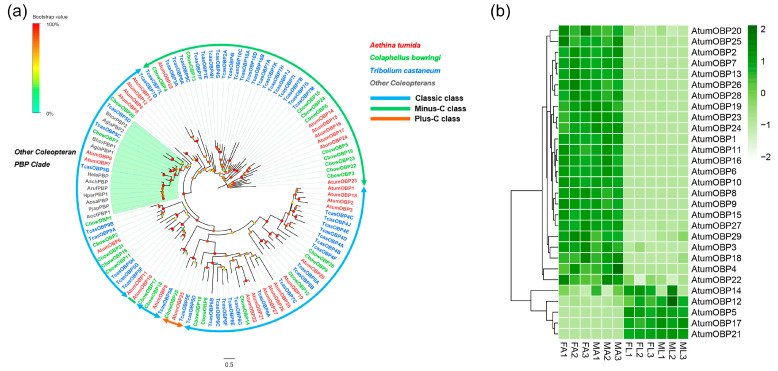
Analyses of putative odorant-binding proteins (OBPs) in *A. tumida*. (**a**) Maximum-likelihood tree of putative OBPs. Circles at the branch nodes represent bootstrap values based on 1000 replicates. Scale bar represents the 0.5 amino acid substitutions per site. Classic OBPs are in blue; Minus-C OBPs in green; Plus-C OBPs in orange. The Coleopteran PBP clade is highlighted as green. (**b**) Heatmap of OBPs based on antennae and forelegs transcriptome data in *A. tumida*. FA, female antennae; MA, male antennae; ML, male forelegs; FL, female forelegs. The transcript abundance was measured by FPKM-values.

Twenty-nine putative OBP-encoding genes (*AtumOBP1*-*29*) were identified. All these candidate genes had a full-length protein ranging from 121 to 303 amino acid, with a secretion signal peptide except *AtumOBP2*, *19,* and *25* (Supplementary Table S2: Sheet 5). Among these AtumOBPs, 20 OBPs showed the Classic motif of six conserved cysteines, eight OBPs were a loss of two otherwise conserved cysteines (Minus-C OBPs), and one was 4–6 additional cysteines (Plus-C OBPs) (Figures S1–S3). A phylogenetic tree was constructed together with OBPs from Coleopterans. Except *AtumOBP10*, AtumOBPs were assigned into Classic OBP, Plus-C OBP and Minus-C OBP based on conserved cysteine residues. Remarkably, *AtumOBP7* and *9* formed a cluster with other Coleopteran pheromone-binding proteins (PBPs). Seven AtumOBPs clustered with other Coleopteran OBPs, while the remaining OBPs formed the sister pairs likely due to species-specific expansion (Figure 5a). All 29 OBPs could be clustered into 2 groups based on the expression levels in different tissues. Cluster analyses indicated that 24 OBP-encoding genes (Cluster 1) share similar expression patterns and were relatively high expressed in FA and MA. The remaining five OBP genes were more highly expressed in the FL and ML (Cluster 2), respectively (Figure 5b). Among the OBP genes expressed abundantly in antennae, *AtumOBP15* was the most abundantly expressed gene (FPKM > 1000), while *AtumOBP10*, *11*, *24*, *25* and *27* were moderately expressed (FPKM > 100). Among the OBP genes expressed highly in forelegs, *AtumOBP21* was the most abundantly expressed OBP (FPKM > 1000).

### 3.7. Candidate Genes Coding for CSPs

**Figure 6:**
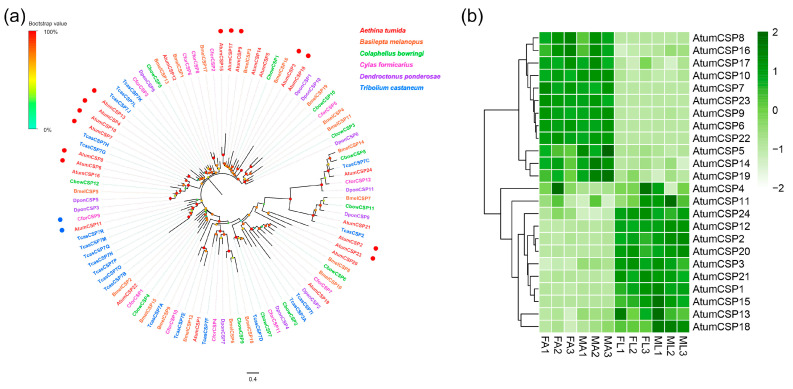
Analyses of putative chemosensory proteins (CSPs) in *A. tumida*. (**a**) Maximum-likelihood tree of putative CSPs. Circles at the branch nodes represent bootstrap values based on 1000 replicates. Scale bar represents the 0.4 amino acid substitutions per site. (**b**) Heatmap of CSPs based on antennae and forelegs transcriptome data in *A. tumida*. FA, female antennae; MA, male antennae; ML, male forelegs; FL, female forelegs. The transcript abundance was measured by FPKM-values.

Twenty-two putative CSP-encoding genes (*AtumCSP1-22*) were identified, with a full-length ORF ranging from 98 to 139 amino acid residues (Supplementary Table S2: Sheet 6). All full-length CSPs had a predicted a signal peptide sequences except for *AtumCSP2* and *17*. All AtumCSPs possessed the four highly conserved cysteine residues (Figure S4). Of these AtumCSPs, three (*AtumCSP1*, *18* and *20*) were located in the same branch along with the orthologous sequences from other Coleopterans, while the remaining CBPs formed the sister pairs (*AtumCSP3/17*, *AtumCSP5/13*, *AtumCSP6/8*) and species-specific expansion (*AtumCSP4/7/10*, *AtumCSP9/14/16*) (Figure 6a). Based on the expression levels in different tissues, all 22 AtumCSPs were clustered to two groups. Group 1 with 12 CSPs exhibited similar expression patterns with relatively high expression levels in FA and MA. Group 2 also contained 10 CSPs that were expressed at high levels in FL and ML (Cluster 2) (Figure 6b). *AtumCSP9* had the highest expression level in antennae, while *AtumCSP11* had the highest expression level in forelegs.

### 3.8. Transcript Levels of SNMPs, OBPs and CSPs

**Figure 7:**
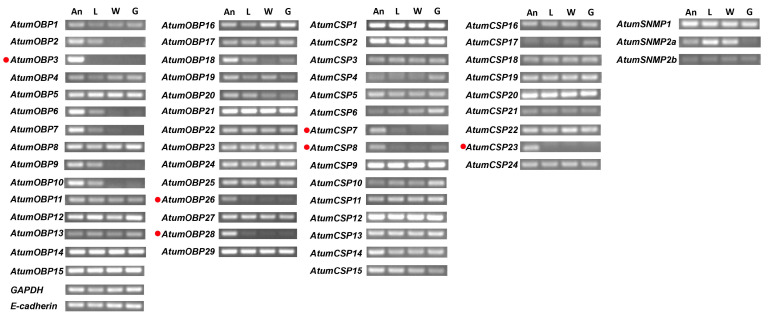
Expression profile of OBPs, CSPs and SNMPs in different tissues. Genes specifically or mainly expressed in antennae are marked with red dots. *GAPDH* and *E-cadherin* genes were used as internal reference to test the integrity of each cDNA template. A, antennae; L, foreleg tarsus; W, wings; G, genitals.

Since SNMPs, OBPs and CSPs in insects were widely expressed in many tissues and might have different roles, we examined their expression levels in four tissues including antennae, foreleg tarsus, wings and genitals using RT-PCR (Figure 7). The result showed that three OBP-encoding genes including *AtumOBP3*, *26,* and *28*, and three CSP-encoding genes including *AtumCSP7*, *8,* and *21*, were abundantly expressed in antennae, whereas other AtumOBPs and AtumCSPs were expressed in multiple body parts. *AtumSNMP1* was also expressed in antennae, forelegs, wings and genitals.

## 4. Discussion

Chemosensory proteins play an important role in insect behavior, including foraging, mating, and oviposition. In this study, we generated transcriptomes from antennae and forelegs of both males and females from *A. tumida*. From these transcriptomes, we identified 130 putative chemosensory genes, including 38 genes coding for ORs, 24 for IRs, 14 for GRs, 3 for SNMPs, 29 for OBPs, and 22 for CSPs. Genetic and phylogenetic analyses were carried out on these genes to examine similarities and differences on related genes.

*AtumOR1* is highly conserved in comparison with Orcos from other Coleopterans. Other genes encoding ORs from *A. tumida* can be scattered into previously defined Coleopteran OR subgroups including subgroups 1–5, 7a and 7b [39,40]. Several expansions specific to *A. tumida* have been found. Expanded OR subgroups in *A. tumida* include subgroup 3 with five members (*AtumOR16/18/19/28/29*), subgroup 7a with eight members (*AtumOR20/32/33/34*), and subgroup 7a with five members (*AtumOR11/12/15/24/31*). Expansion of OR gene subgroups specific to an insect species has also been found in other insect species, and it is generally acknowledged a strategy for a species to adapt to a distinct ecological niche [39,40]. Studies on the expanded subgroups may lead to targets for semiochemical discovery that could be used to manipulate insect behaviors for pest control. Transcriptome analysis showed all the ORs were up regulated in the antennae as compared to samples from forelegs. No significant difference in gene expression levels of ORs was found between antennae from males and females. This observation is consistent with what has been reported in the literature; namely, no differences in sexually dimorphic behavior in response to semiochemicals have been reported between males and females of Nitidulidae species including *A. tumida* [42]. This might suggest that *A. tumida* uses vibrations to mediate sexual communication among adults rather than chemical signals among Coleoptera families including Anobiidae [43], Tenebrionidae [44], Cerambycidae [45], and Curculionidae [46].

IRs are the most ancient chemoreceptors for odor sensation and taste sensation [4,47,48,49,50,51], as well as for thermosensation and hygrosensation [52,53,54]. The three genes encoding IR co-receptors *AtumIR76b*, *AtumIR25a* and *IR8a*, and 13 antennal IR-encoding genes including *AtumIR21a*, *IR41a*, *IR60a*, *IR68a*, *IR75c*, *IR75q1*, *IR75q2*, *IR75q3*, *IR75s,* and *IR93a* identified here have orthologs in other Coleopterans. Based on data from *D. melanogaster*, *IR64a* is sensitive to acetate, propionate and butyrate [55]. *IR41a*, together with *IR76b*, mediate long-range attraction to odor [51]. *IR21a*, together with *IR25a*, mediate behavioral responses to cool conditions [53]. *IR93a* and *IR68a* mediate behavioral responses to both temperature and moisture [52,54]. *IR75q1* and *IR75q2* are necessary for moths to conduct acetic acid preference, with *IR75q1* recognizing acetic acid and *IR75q2* amplifying sensitivity [56]. The IR orthologs in *A. tumida* might play similar roles in sensory perception due to their high similarity. At present, the function of the *IR60a* remains to be determined and further investigation is needed to reveal its function. As expected, transcriptome analysis also showed all the antennal IRs had higher expression in the antennae as compared to the forelegs, and no sex-biased expression was found. This suggests that IR as an ancient chemosensory receptor family [10] function as chemoreceptors (detecting odorants and tastants), thermoreceptors, or hygroreceptors that might be of general importance for insects, regardless of sex.

OBPs are commonly regarded as solubilizers and carriers of odorants [16] and can enhance the sensitivity of olfactory receptors to odorants, such as host chemicals and pheromones [57,58,59,60,61]. However, large numbers of OBPs are not restricted to olfactory organs and may have various other roles [16]. According to the RT-PCR result, among the identified OBPs here, three Classic OBP-encoding genes (*AtumOBP3*, *26* and *28*) were highly expressed in antennae, suggesting that they may play roles in olfactory perception. The two Classic OBPs, *AtumOBP7* and *9*, formed a cluster with other Coleopteran PBPs [62]. We hypothesize that *AtumOBP7* and *9* are putative PBPs in *A. tumida*. Similar to OBPs, CSPs are also postulated to function as carriers of odorant molecules [62]. Among the identified AtumCSPs, three CSP-encoding genes, namely *AtumCSP7*, *8*, and *21*, were highly expressed in antennae and their roles are likely involved in olfaction. Similar to the OR family, sex-biased expression was not detected in the antenna-predominant OBPs analyzed by RNA-seq. The expression profile of OBPs might function in the detection and discrimination of host volatiles or pheromone that elicit aggregation behaviors in both sexes [63]. RT-PCR showed all OBPs (24) and CSPs (12) were expressed in antennae which were consistent with the transcriptome. However, the numbers of genes encoding antenna-specific OBPs (3) and CSPs (3) in *A. tumida* are much smaller than most other Coleopterans [64,65,66]. The reduction in the numbers of OBP- and CSP-encoding genes in *A. tumida* may be due to host adaptation since *A. tumida* have relatively narrow host ranges. *A. tumida* is primarily a parasite of bee colonies. Further study on the function of the antenna specific OBPs and CSPs in *A. tumida* is needed to explore their functions.

## 5. Conclusions

In the present study, we generated transcriptomes from antennae and forelegs of both males and females from *A. tumida*. From these transcriptomes, we identified 132 putative chemosensory genes, including 38 ORs, 24 IRs, 14 GRs, 3 SNMPs, 29 OBPs, and 22 CSPs. Furthermore, 3 OBPs (*AtumOBP3*, *26* and *28*) and 3 CSPs (*AtumCSP7*, *8* and *21*) were identified to be highly expressed in antennae. Our results might provide a foundation for the further study of olfactory function and the biological control of the small hive beetle.

## Data Availability

All data produced from this study are included in this published paper.

## Supplementary Materials

The following are available online at https://www.mdpi.com/article/10.3390/insects12080661/s1, Figure S1: Multiple amino acid alignment of the predicted Classic OBPs, Figure S2: Multiple amino alignment of the predicted Minus-C OBPs, Figure S3: Multiple amino alignment of the predicted Plus-C OBPs, Figure S4: Multiple amino alignment of the predicted CSPs, Table S1: List of chemosensory genes in Coleopteran species, Table S2: List of the identified chemosensory genes and reference genes in *A. tumida*, and detailed genetic characteristics, best matches in NCBI-nr database and protein domains found in each petal module, Table S3: Amino acid sequences used for phylogenetic analyses, Table S4: The estimated expression levels (FPKM value) of chemosensory genes, Table S5: Primer pairs used for RT-PCR, Table S6: An overview of the sequencing and assembly of *A. tumida*.

## References

[1] Sato K., Touhara K. (2009). Insect olfaction: Receptors, signal transduction, and behavior. Results Probl. Cell Differ..

[2] Leal W.S. (2013). Odorant reception in insects: Roles of receptors, binding proteins, and degrading enzymes. Annu. Rev. Entomol..

[3] Joseph R.M., Carlson J.R. (2015). Drosophila chemoreceptors: A molecular interface between the chemical world and the brain. Trends Genet..

[4] Benton R., Vannice K.S., Gomez-Diaz C., Vosshall L.B. (2009). Variant ionotropic glutamate receptors as chemosensory receptors in Drosophila. Cell.

[5] Touhara K., Vosshall L.B. (2009). Sensing odorants and pheromones with chemosensory receptors. Annu. Rev. Physiol..

[6] Vogt R.G., Riddiford L.M. (1981). Pheromone binding and inactivation by moth antennae. Nature.

[7] Sato K., Pellegrino M., Nakagawa T., Nakagawa T., Vosshall L.B., Touhara K. (2008). Insect olfactory receptors are heteromeric ligand-gated ion channels. Nature.

[8] Croset V., Rytz R., Cummins S.F., Budd A., Brawand D., Kaessmann H., Gibson T.J., Benton R. (2010). Ancient protostome origin of chemosensory ionotropic glutamate receptors and the evolution of insect taste and olfaction. PLoS Genet..

[9] Kwon J.Y., Dahanukar A., Weiss L.A., Carlson J.R. (2007). The molecular basis of CO2 reception in Drosophila. Proc. Natl. Acad. Sci. USA.

[10] Sato K., Tanaka K., Touhara K. (2011). Sugar-regulated cation channel formed by an insect gustatory receptor. Proc. Natl. Acad. Sci. USA.

[11] Benton R., Vannice K.S., Vosshall L.B. (2007). An essential role for a CD36-related receptor in pheromone detection in Drosophila. Nature.

[12] Jin X., Ha T.S., Smith D.P. (2008). SNMP is a signaling component required for pheromone sensitivity in Drosophila. Proc. Natl. Acad. Sci. USA.

[13] Li Z., Ni J.D., Huang J., Montell C. (2014). Requirement for Drosophila SNMP1 for rapid activation and termination of pheromone-induced activity. PLoS Genet..

[14] Gomez-Diaz C., Bargeton B., Abuin L., Bukar N., Reina J.H., Bartoi T., Graf M., Ong H., Ulbrich M.H., Masson J.F. (2016). A CD36 ectodomain mediates insect pheromone detection via a putative tunnelling mechanism. Nat. Commun..

[15] Vogt R.G., Miller N.E., Litvack R., Fandino R.A., Sparks J., Friedman R., Dickens J.C. (2009). The insect SNMP gene family. Insect Biochem. Mol. Biol..

[16] Pelosi P., Iovinella I., Felicioli A., Dani F.R. (2014). Soluble proteins of chemical communication: An overview across arthropods. Front. Physiol..

[17] Ban L., Napolitano E., Serra A., Zhou X., Iovinella I., Pelosi P. (2013). Identification of pheromone-like compounds in male reproductive organs of the oriental locust Locusta migratoria. Biochem. Biophys. Res. Commun..

[18] Iovinella I., Bozza F., Caputo B., Della T.A., Pelosi P. (2013). Ligand-binding study of Anopheles gambiae chemosensory proteins. Chem. Senses..

[19] Zhang T., Wang W., Zhang Z., Zhang Y., Guo Y. (2013). Functional characteristics of a novel chemosensory protein in the cotton bollworm Helicoverpa armigera (Hubner). J. Integr. Agric..

[20] Zhang Y.N., Ye Z.F., Yang K., Dong S.L. (2014). Antenna-predominant and male-biased CSP19 of Sesamia inferensis able to bind the female sex pheromones and host plant volatiles. Gene.

[21] Neumann P., Pettis J.S., Schäfer M.O. (2016). Quo vadis Aethina tumida? Biology and control of small hive beetles. Apidologie.

[22] Toufailia H.A., Alves D.A., Bená D.D.C., Bento J.M.S., Iwanicki N.S.A., Cline A.R., Ellis J.D., Ratnieks F.L.W. (2017). First record of small hive beetle, Aethina tumida Murray, in South America. J. Apic. Res..

[23] Idrissou F.O., Huang Q., Yanez O., Neumann P. (2019). International beeswax trade facilitates small hive beetle invasions. Sci. Rep..

[24] Neumann P., Ellis J.D. (2008). The small hive beetle (Aethina tumida Murray, Coleoptera: Nitidulidae): Distribution, biology and control of an invasive species. J. Apic. Res..

[25] Graham J.R., Ellis J.D., Carroll M.J., Teal P.E.A. (2011). Aethina tumida (Coleoptera: Nitidulidae) attraction to volatiles produced by Apis mellifera (Hymenoptera: Apidae) and Bombus impatiens (Hymenoptera: Apidae) colonies. Apidologie.

[26] Guzman L.I.D., Frake A.M., Rinderer T.E., Arbogast R.T. (2011). Effect of height and color on the efficiency of pole traps for Aethina tumida (Coleoptera: Nitidulidae). J. Chem. Ecol..

[27] Suazo A., Torto B., Teal P.E.A., Tumlinson J.H. (2003). Response of the small hive beetle (Aethina tumida) to honey bee (Apis mellifera) and beehive-produced volatiles. Apidologie.

[28] Jayanthi K.P., Kempraj V., Aurade R.M., Roy T.K., Shivashankara K.S., Verghese A. (2014). Computational reverse chemical ecology: Virtual screening and predicting behaviorally active semiochemicals for Bactrocera dorsalis. BMC Genom..

[29] Choo Y.M., Xu P., Hwang J.K., Zeng F., Tan K., Bhagavathy G., Chauhan K.R., Leal W.S. (2018). Reverse chemical ecology approach for the identification of an oviposition attractant for Culex quinquefasciatus. Proc. Natl. Acad. Sci. USA.

[30] Venthur H., Zhou J.J. (2018). Odorant receptors and Odorant-Binding proteins as insect pest control targets: A comparative analysis. Front. Physiol..

[31] Klijnstra J., Roessingh P. (1986). Perception of the oviposition deterring pheromone by tarsal and abdominal contact chemoreceptors in Pieris brassicae. Entomol. Exp. Appl..

[32] Ling F., Dahanukar A., Weiss L.A., Kwon J.Y., Carlson J.R. (2014). The molecular and cellular basis of taste coding in the legs of Drosophila. J. Neurosci..

[33] Quevillon E., Silventoinen V., Pillai S., Harte N., Mulder N., Apweiler R., Lopez R. (2005). InterProScan: Protein domains identifier. Nucleic Acids Res..

[34] Sievers F., Wilm A., Dineen D., Gibson T.J., Karplus K., Li W., Lopez R., Mcwilliam H., Remmert M., Söding J. (2011). Fast, scalable generation of high-quality protein multiple sequence alignments using Clustal Omega. Mol. Syst. Biol..

[35] Price M.N., Dehal P.S., Arkin A.P. (2010). FastTree 2-approximately maximum-likelihood trees for large alignments. PLoS ONE.

[36] Mortazavi A., Williams B.A., McCue K., Schaeffer L., Wold B. (2008). Mapping and quantifying mammalian transcriptomes by RNA-Seq. Nat. Methods.

[37] Love M.I., Huber W., Anders S. (2014). Moderated estimation of fold change and dispersion for RNA-seq data with DESeq2. Genome Biol..

[38] Untergasser A., Cutcutache I., Koressaar T., Ye J., Faircloth B.C., Remm M., Rozen S.G. (2012). Primer3-new capabilities and interfaces. Nucleic Acids Res..

[39] Engsontia P., Sanderson A.P., Cobb M., Walden K.K., Robertson H.M., Brown S. (2008). The red flour beetle’s large nose: An expanded odorant receptor gene family in Tribolium castaneum. Insect Biochem. Mol. Biol..

[40] Mitchell R.F., Hughes D.T., Luetje C.W., Millar J.G., Soriano-Agaton F., Hanks L.M., Robertson H.M. (2012). Sequencing and characterizing odorant receptors of the cerambycid beetle Megacyllene caryae. Insect Biochem. Mol. Biol..

[41] Miyamoto T., Slone J., Song X., Amrein H. (2012). A fructose receptor functions as a nutrient sensor in the Drosophila brain. Cell.

[42] Takanashi T., Uechi N., Tatsuta H. (2019). Vibrations in hemipteran and coleopteran insects: Behaviors and application in pest management. Appl. Entomol. Zool..

[43] Goulson D., Birch M.C., Wyatt T.D. (1994). Mate location in the deathwatch beetle, Xestobium rufovillosum De Geer (Anobiidae): Orientation to substrate vibrations. Anim. Behav..

[44] Kiyotake H., Matsumoto H., Nakayama S., Sakai M., Miyatake T., Ryuda M., Hayakawa Y. (2014). Gain of long tonic immobility behavioral trait causes the red flour beetle to reduce anti-stress capacity. J. Insect Physiol..

[45] Tsubaki R., Hosoda N., Kitajima H., Takanashi T. (2014). Substrate-borne vibrations induce behavioral responses in the leaf-dwelling cerambycid, Paraglenea fortunei. Zoolog. Sci..

[46] Fleming A., Lindeman A.A., Carroll A.L., Yack J. (2013). Acoustics of the mountain pine beetle (Dendroctonus ponderosae) (Curculionidae, Scolytinae): Sonic, ultrasonic, and vibration characteristics. Can. J. Zool..

[47] Ai M., Min S., Grosjean Y., Leblanc C., Bell R., Benton R., Suh G.S. (2010). Acid sensing by the Drosophila olfactory system. Nature.

[48] Silbering A.F., Rytz R., Grosjean Y., Abuin L., Ramdya P., Jefferis G.S.X.E., Benton R. (2011). Complementary function and integrated wiring of the evolutionarily distinct Drosophila olfactory subsystems. J. Neurosci..

[49] Kain P., Boyle S.M., Tharadra S.K., Guda T., Christine P., Dahanukar A., Ray A. (2013). Odour receptors and neurons for DEET and new insect repellents. Nature..

[50] Koh T.W., He Z., Gorur-Shandilya S., Menuz K., Larter N.K., Stewart S., Carlson J.R. (2014). The Drosophila IR20a clade of ionotropic receptors are candidate taste and pheromone receptors. Neuron.

[51] Hussain A., Zhang M., Ucpunar H.K., Svensson T., Quillery E., Gompel N., Ignell R., Grunwald K.I. (2016). Ionotropic chemosensory receptors mediate the taste and smell of polyamines. PLoS Biol..

[52] Knecht Z.A., Silbering A.F., Ni L., Klein M., Budelli G., Bell R., Abuin L., Ferrer A.J., Samuel A.D., Benton R. (2016). Distinct combinations of variant ionotropic glutamate receptors mediate thermosensation and hygrosensation in Drosophila. Elife.

[53] Ni L., Klein M., Svec K.V., Budelli G., Chang E.C., Ferrer A.J., Benton R., Samuel A.D., Garrity P.A. (2016). The Ionotropic Receptors IR21a and IR25a mediate cool sensing in Drosophila. Elife.

[54] Knecht Z.A., Silbering A.F., Cruz J., Yang L., Croset V., Benton R., Garrity P.A. (2017). Ionotropic Receptor-dependent moist and dry cells control hygrosensation in Drosophila. Elife.

[55] Ai M., Blais S., Park J.Y., Min S., Neubert T.A., Suh G.S. (2013). Ionotropic glutamate receptors IR64a and IR8a form a functional odorant receptor complex in vivo in Drosophila. J. Neurosci..

[56] Tang R., Jiang N., Ning C., Li G., Huang L., Wang C. (2020). The olfactory reception of acetic acid and ionotropic receptors in the Oriental armyworm, Mythimna separata Walker. Insect Biochem. Mol. Biol..

[57] Xu P.X., Atkinson R., Jones D., Smith D.P. (2005). Drosophila OBP LUSH is required for activity of pheromone-sensitive neurons. Neuron.

[58] Gomez-Diaz C., Reina J.H., Cambillau C., Benton R. (2013). Ligands for pheromone-sensing neurons are not conformationally activated odorant binding proteins. PLoS Biol..

[59] Chang H., Liu Y., Yang T., Pelosi P., Dong S., Wang G.R. (2015). Pheromone binding proteins enhance the sensitivity of olfactory receptors to sex pheromones in Chilo suppressalis. Sci. Rep..

[60] Wu Z., Lin J., Zhang H., Zeng X. (2018). BdorOBP83a-2 mediates responses of the oriental fruit fly to semiochemicals. Front. Physisiol..

[61] Xiao S., Sun J.S., Carlson J.R. (2019). Robust olfactory responses in the absence of odorant binding proteins. Elife.

[62] Wen X., Wang Q., Gao P., Wen J. (2018). Identification and comparison of chemosensory genes in the antennal transcriptomes of *Eucryptorrhynchus scrobiculatus* and *E. brandti* fed on *Ailanthus altissima*. Front. Physiol..

[63] Stuhl C.J., Teal P.E.A. (2020). Identification of an aggregation pheromone from the small hive beetle (Coleoptera: Nitidulidae). BioRxiv.

[64] Wu Z., Bin S., He H., Wang Z., Li M., Lin J. (2016). Differential expression analysis of chemoreception genes in the striped flea beetle Phyllotreta striolata using a transcriptomic approach. PLoS ONE.

[65] Bin S.Y., Qu M.Q., Li K.M., Peng Z.Q., Wu Z.Z., Lin J.T. (2017). Antennal and abdominal transcriptomes reveal chemosensory gene families in the coconut hispine beetle. Sci. Rep..

[66] Bin S.Y., Qu M.Q., Pu X.H., Wu Z.Z., Lin J.T. (2017). Antennal transcriptome and expression analyses of olfactory genes in the sweetpotato weevil Cylas formicarius. Sci. Rep..

