# Supplementary figures and images for: Identification and Expression Profile of Chemosensory Genes in the Small Hive Beetle Aethina tumida
Source: Insects. 2021 Jul 21;12(8):661. doi: 10.3390/insects12080661 (PMC8396569; doi:10.3390/insects12080661)

Fig. S1

Sequence Logo

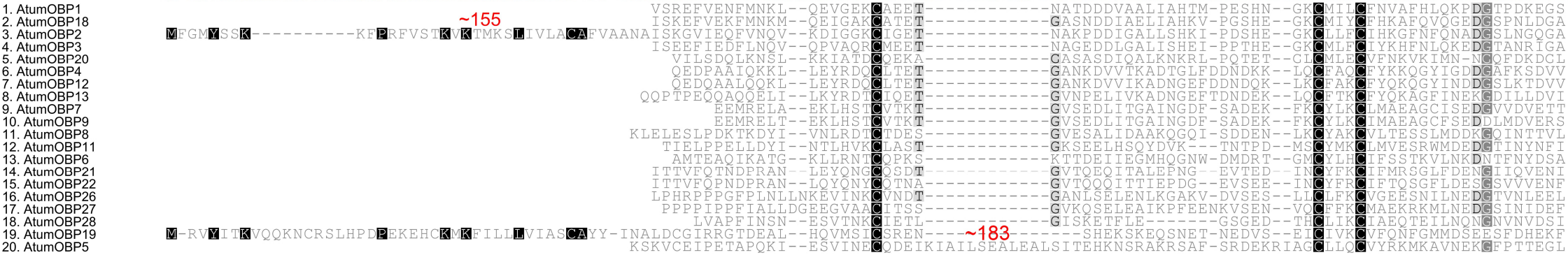

Sequence Logo

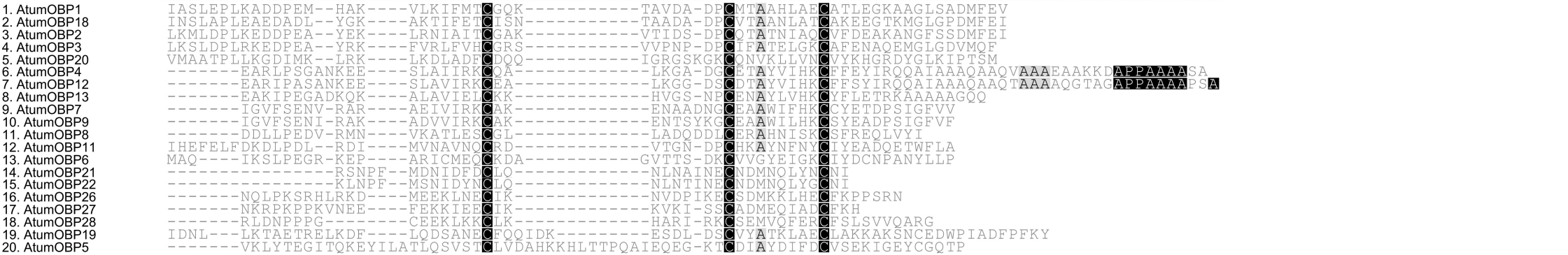

C4

C5

C6

Fig. S2

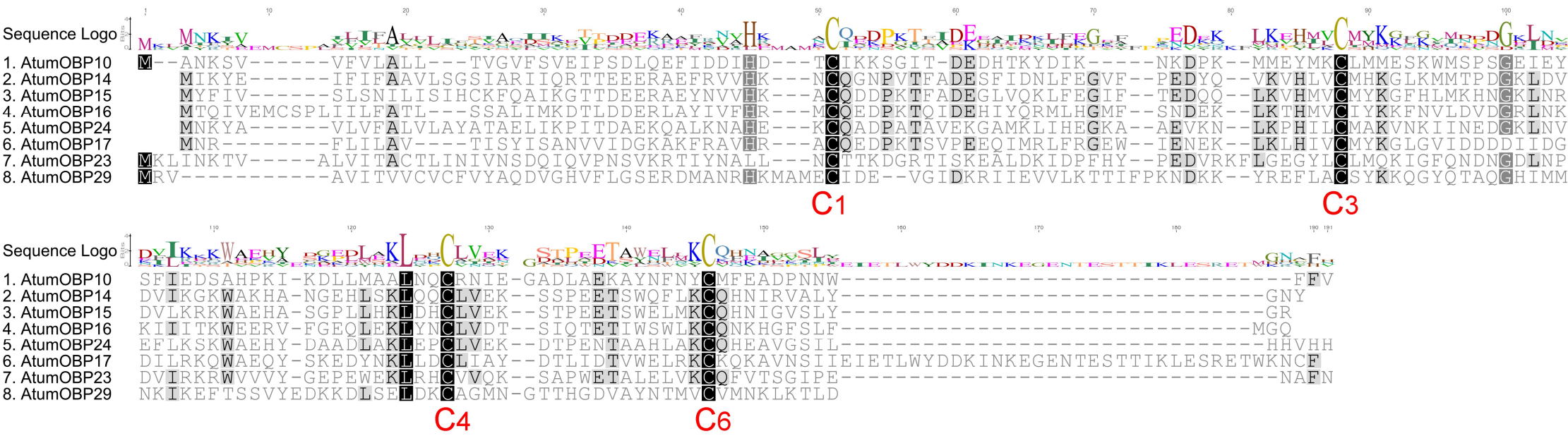

Fig. S3

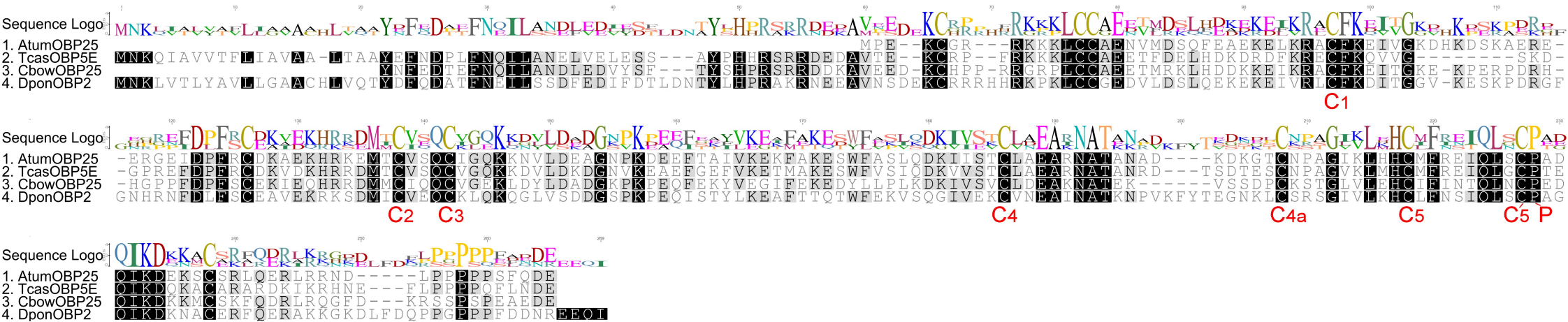

Fig. S4

Sequence Logo

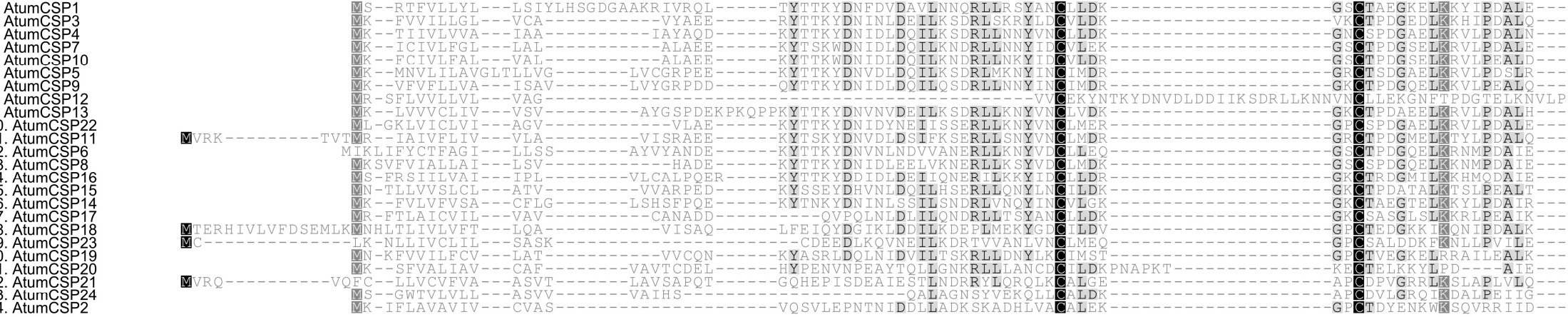

C1

C2

Sequence Logo

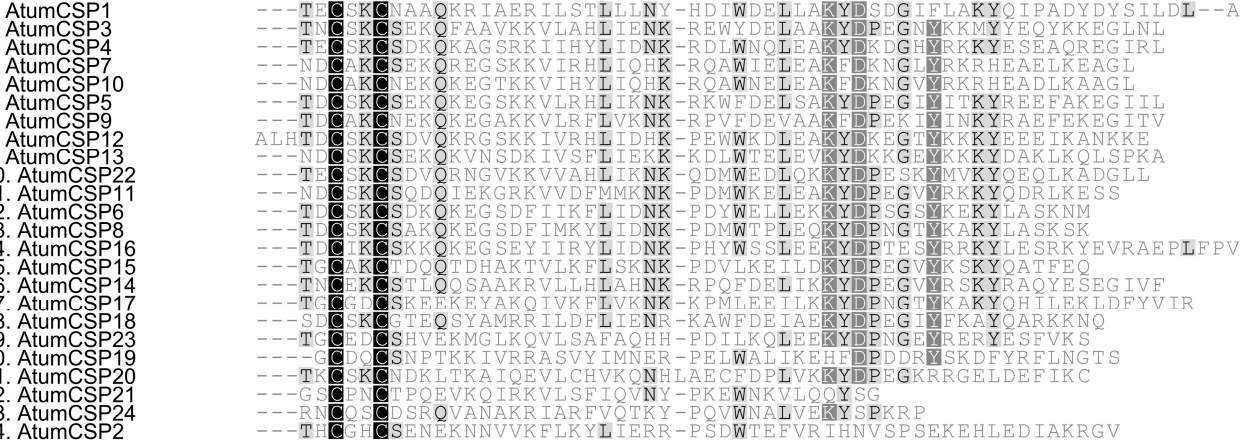

C3 C4

Supplement: Supplementary file 1 [file insects-12-00661-s001.zip › Supplemental information/Supplementary figures.pdf]
